# Nusinersen treatment and cerebrospinal fluid neurofilaments: An explorative study on Spinal Muscular Atrophy type 3 patients

**DOI:** 10.1111/jcmm.14939

**Published:** 2020-02-07

**Authors:** Irene Faravelli, Megi Meneri, Domenica Saccomanno, Daniele Velardo, Elena Abati, Delia Gagliardi, Valeria Parente, Lucia Petrozzi, Dario Ronchi, Nino Stocchetti, Edoardo Calderini, Grazia D’Angelo, Giovanna Chidini, Edi Prandi, Giulia Ricci, Gabriele Siciliano, Nereo Bresolin, Giacomo Pietro Comi, Stefania Corti, Francesca Magri, Alessandra Govoni

**Affiliations:** ^1^ Dino Ferrari Centre Department of Pathophysiology and Transplantation (DEPT) University of Milan Milan Italy; ^2^ Fondazione IRCCS Ca' Granda Ospedale Maggiore Policlinico Neurology Unit Milan Italy; ^3^ Neurological Clinics Department of Clinical and Experimental Medicine University of Pisa Pisa Italy; ^4^ Fondazione IRCCS Ca' Granda Ospedale Maggiore Policlinico Department of Physiopathology and Transplantation Milan University and Neuro ICU Milan Italy; ^5^ Pediatric Intensive Care Unit Mother & Child Anaesthesia and Emergency Departement Fondazione IRCCS Ca’ Granda Ospedale Maggiore Policlinico Milan Italy; ^6^ Scientific institute IRCCS E Medea Lecco Italy; ^7^ Neuromuscular and Rare Disease Unit Fondazione IRCCS Ca' Granda Ospedale Maggiore Policlinico Milan Italy

**Keywords:** neurofilaments, Nusinersen, pharmacodynamics biomarker, spinal muscular atrophy

## Abstract

The antisense oligonucleotide Nusinersen has been recently licensed to treat spinal muscular atrophy (SMA). Since SMA type 3 is characterized by variable phenotype and milder progression, biomarkers of early treatment response are urgently needed. We investigated the cerebrospinal fluid (CSF) concentration of neurofilaments in SMA type 3 patients treated with Nusinersen as a potential biomarker of treatment efficacy. The concentration of phosphorylated neurofilaments heavy chain (pNfH) and light chain (NfL) in the CSF of SMA type 3 patients was evaluated before and after six months since the first Nusinersen administration, performed with commercially available enzyme‐linked immunosorbent assay (ELISA) kits. Clinical evaluation of SMA patients was performed with standardized motor function scales. Baseline neurofilament levels in patients were comparable to controls, but significantly decreased after six months of treatment, while motor functions were only marginally ameliorated. No significant correlation was observed between the change in motor functions and that of neurofilaments over time. The reduction of neurofilament levels suggests a possible early biochemical effect of treatment on axonal degeneration, which may precede changes in motor performance. Our study mandates further investigations to assess neurofilaments as a marker of treatment response.

## 1 INTRODUCTION

Spinal muscular atrophy (SMA) is an autosomal recessive neuromuscular disease caused by mutations in the Survival Motor Neuron 1 (SMN1) gene. The most severe form of this disease, SMA type 1, becomes evident in the firsts months of life and is currently the most common genetic cause of death in infancy.^1^ On the other hand, individuals affected by SMA type 3 usually display a milder phenotype and a more indolent disease progression; despite this, most patients lose their ability to walk independently in adulthood.^2^ The main determinant of these different outcome relates to the variable copy number of the gene SMN2^3^: Most of the SMN protein that derives from this paralogous gene is rapidly degraded, but up to 10% of the total protein product is functional and may in part compensate the complete lack of SMN1. However, other factors are likely to play a role in determining the clinical phenotype.^4^

Nusinersen, the first marketed disease‐modifying drug for SMA,^5^ is an antisense oligonucleotide that binds to the SMN2 pre‐mRNA downstream of exon 7, leading to the translation of a fully functional SMN protein. Nusinersen is administered intrathecally by lumbar puncture and has been recently licensed in Europe to treat SMA in each country with specific indications for the different types of SMA.^6^ Results of clinical trials have shown a dramatic clinical and laboratory response after Nusinersen administration in SMA type 1 patients,^7^ while recent studies on late‐onset SMA type 3 provided less striking, albeit encouraging results.^8^ Indeed, SMA type 3 patients display a highly variable phenotypic spectrum of disease and, due to the relatively milder progression typical of this subset of patients, clinical differences may theoretically become evident only after prolonged treatment. Considering the scant data available on Nusinersen efficacy in this population and the high cost of this intrathecally administered drug, robust biomarkers of early treatment response are required to identify patients who could benefit from treatment.

Phosphorylated neurofilament heavy chain (pNfH) and neurofilament light chain (NfL) have been reproducibly associated with axonal degeneration,^9^ and levels of these biomarkers tend to increase in both cerebrospinal fluid (CSF) and plasma/serum of patients affected by motor neuron diseases (MNDs). As a result, neurofilaments have been increasingly recognized as a useful biomarker for the diagnosis of amyotrophic lateral sclerosis, and their concentration in the CSF is affected by disease duration, rate of progression and involvement of first‐ and second‐motor neurons.^10^

A recent study suggested that plasma pNfH could be exploited as a biomarker of treatment response to Nusinersen in SMA type 1 patients^7^: increased pNfH levels at baseline with respect to 34 controls showed a rapid and substantial decline after only two months from treatment start, reaching a 90.1% relative change at 10 months. In the present study, we aimed to assess the levels and changes over time of pNfH and NfL in the CSF of type 3 SMA patients treated with intrathecal Nusinersen, to explore their use as a potential biomarkers of treatment response.

## 2 MATERIALS AND METHODS

### 2.1 Clinical evaluation

Spinal muscular atrophy type 3 patients were recruited at the Neurology Unit of Fondazione IRCCS Ca’ Granda Ospedale Maggiore Policlinico di Milano and at the Neurology Unit of Azienda Ospedaliero‐Universitaria of Pisa. Patients were evaluated with a 6‐month follow‐up, starting before the first Nusinersen administration and on the following treatment days (at 2 weeks and at one, 2 and 6 months from the first treatment, according to the national Agenzia Italiana del Farmaco—AIFA guidelines). All adult and paediatric patients were regularly evaluated over time with standardized motor function scales: Hammersmith Functional Motor Scale Expanded (HFMSE) and 6 Minute Walking Test (6MWT) for ambulant patients. For reference, a single SMA type 1 patient undergoing the same procedures and evaluations was recruited from one of the two institutions.

The study was conducted in accordance with the Declaration of Helsinki and followed ICH GCP guidelines. All subjects and the parents of minors provided written informed consent for the collection, storage and analysis of biological materials according to the disposition of the local ethics committees.

### 2.2 CSF analysis

Cerebrospinal fluid samples were collected right before the intrathecal administration of Nusinersen. Analyses were performed on samples obtained on the first (pre‐treatment) and on the last administration (6 months). CSF analyses were also performed on selected samples from subjects who underwent lumbar puncture during a diagnostic procedure for unconfirmed clinical suspicions and who were ultimately diagnosed with psychosomatic diseases. Patients with different neurological conditions (excluding neurodegenerative/motor neuron disorders) with an age range comparable to SMA patients were also included. CSF pNfH and NfL levels were measured performed with commercially available enzyme‐linked immunosorbent assay (ELISA) kits (pNfH: Euroimmun, Lubeck, Germany—NfL: UmanDiagnostics AB, Umea, Sweden) according to the manufactures’ instructions.

### 2.3 Statistical analysis

Baseline characteristics were analysed through descriptive statistics, continuous variables were reported as mean ± SD or median [IQR], as appropriate. Categorical variables were represented as numbers and percentages. Normality of distributions was visually inspected by box‐plot representation and formally assessed with the D'Agostino‐Pearson's test. For both pNfH and NfL, values below the lower limit of quantification were approximated to half of the concentration of the lowest calibrator (50 pg/mL and 0.0625 ng/mL for NfL and NfH, respectively). Between‐group comparisons were performed with the Mann‐Whitney test. Within‐group comparisons over time were carried out by means of paired *t* test or Wilcoxon matched‐pairs signed‐rank test, as appropriate. Associations between variables were assessed with the nonparametric Spearman's correlation coefficient. Statistically significant differences were assumed at 5% level of probability, and all statistical tests were two‐tailed. All analyses were performed with GraphPad Prism version 8 (GraphPad Software Inc).

## 3 RESULTS

### 3.1 Clinical assessment

A total of 12 patients diagnosed with SMA type 3 were enrolled in the study. The age of first treatment ranged from a minimum of nine to a maximum of 74 years, with nine patients (75%) >18 years old. The minimum disease duration at the start of treatment is 7.5 years, while the maximum duration is 32 years. Baseline characteristics are summarized in Table 1. Two patients used wheelchairs since the age of 18 and 29 years, and another two had mild respiratory involvement due to restrictive pulmonary disease and obstructive sleep‐apnoea syndrome respectively. Ten patients were ambulant, but one of them needed support and was able to walk only for few steps, while another one underwent minor orthopaedic surgery at the beginning of the observation period, thus only eight patients were able to perform the 6WMT both at baseline and at the end of the follow‐up. A single patient with SMA type 1, who started therapy at the age of 3 months, was also included as reference. All Nusinersen administrations were well‐tolerated, and the only adverse event reported was headache after lumbar puncture in 6 (50%) subjects after the first drug infusion.

**Table 1:** Baseline clinic characteristics of SMA type 3 patients

Characteristic	SMA III (n = 12)
Female Sex – n° (%)	4 (33.3%)
Age at Onset – years	5.5 [2.3‐14.8]
Age at Treatment – years	28.5 [15.0‐34.8]
Disease Duration – years	20.1 ± 10.0
SMN2 Copy Numbers – n° (%)	
2	1 (8.3%)
3	7 (58.3%)
4	4 (33.3%)
Ambulant – n° (%)	10 (83.3%)
Respiratory involvement – n° (%)	2 (16.7%)
HFMSE (score)	40.8 ± 13.5
6MWT (metres)	304 ± 126

Note

Categorical data expressed as number (%), continuous data reported as mean ± SD or median [IQR], as appropriate.

**Figure 1:**
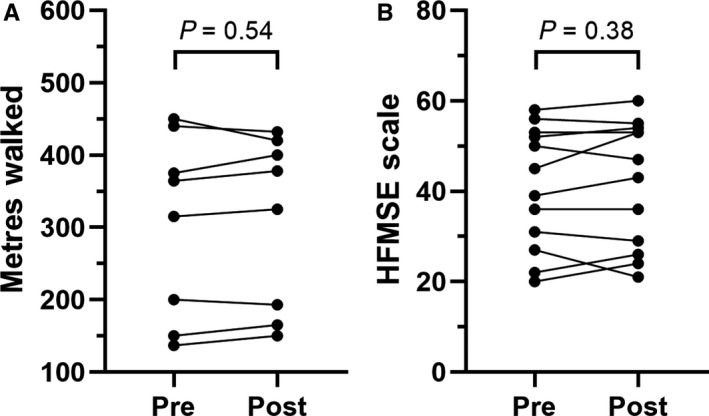
Evaluation of standardized motor scales in SMA type 3 patients. Individual scores at the 6 min walking test (A) and at the Hammersmith Functional Motor Scale Expanded (B) at baseline (pre) and after five injections (post) of Nusinersen

Motor functions were not significantly modified by Nusinersen in SMA type 3 patients during the 6‐month follow‐up, with a non‐significant trend towards improved performances in the 6‐minutes walking test (+4.0 ± 17.7 metres, *P* = .54) and in the HFMSE score (+1.0 ± 3.8 points, *P* = .38) compared to baseline (Figure 1A and 1, respectively).

### 3.2 Neurofilaments in the CSF

**Figure 2:**
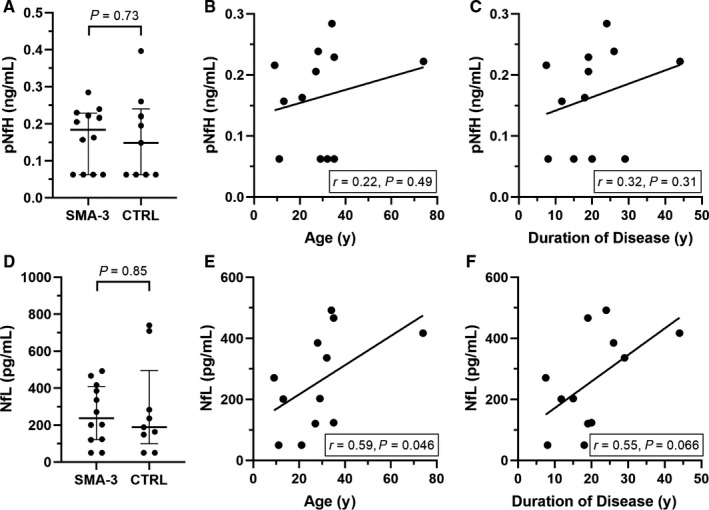
Cerebrospinal fluid (CSF) concentrations of phosphorylated neurofilament heavy chain (pNfH) and neurofilament light chain (NfL). Levels of pNfH (A) and NfL (D) in the CSF of SMA type 3 patients (SMA‐3) and controls (median [IQR]). Correlation between pNfH levels and either age at treatment (B) or duration of disease (C). The same analyses for NfL are also displayed (E‐F). 4 of 12 pNfH (A) and 2 of 12 NfL (D) values at baseline were below the detection limit

pNfH and NfL levels dosed at baseline in the CFS of SMA type 3 patients were comparable to those observed in controls (n = 9, age: 29.0 [22.0‐54.5] year‐old) and were far below the average values described in patients affected by either SMA type 1 (6.874 ng/mL and >10 000 pg/mL, respectively) or amyotrophic lateral sclerosis (5.270 ng/mL and 2961 pg/mL^11^) (Figure 2A and 2). The baseline concentration of neurofilaments in our study was below the lower limit of quantification in a significant proportion of patients (4/12 and 2/12 for pNfH and NfL respectively). In these cases, lower limit of quantification was approximated to half of the concentration of the lowest calibrator (50 pg/mL and 0.0625 ng/mL for NfL and NfH, respectively).

No significant correlation was observed between pNfH and either age at treatment or duration of the disease (Figure 2B and 2). On the other hand, a positive correlation was found between NfL and age at treatment (*r* = 0.59, *P* = .046) along with a marginally significant correlation between NfL and duration of the disease (*r* = 0.55, *P* = .066) (Figure 2E and 2).

**Figure 3:**
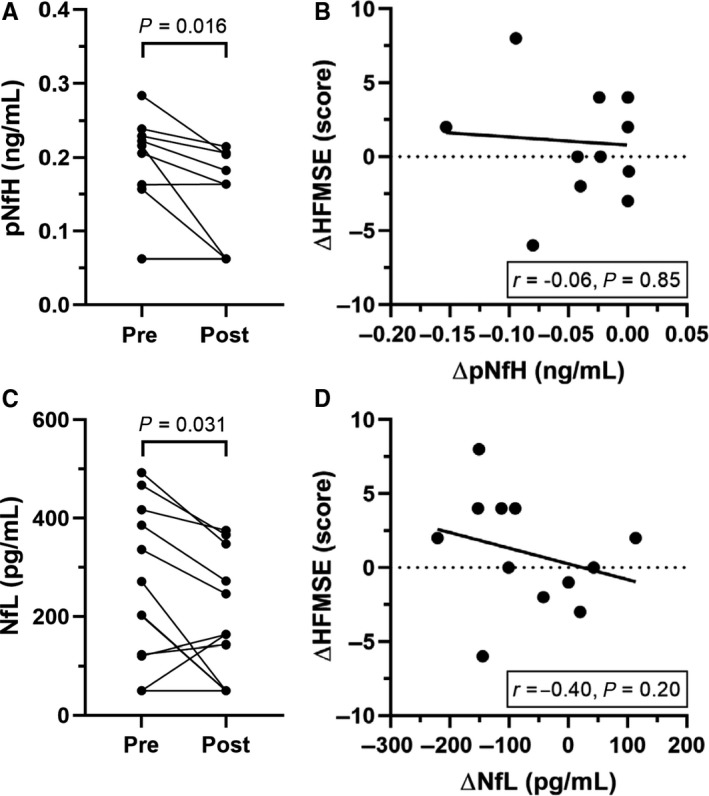
Cerebrospinal fluid (CSF) concentrations of phosphorylated neurofilament heavy chain (pNfH) and neurofilament light chain (NfL) at baseline and after treatment with Nusinersen. Individual CSF pNFH levels (A) and NfL levels (C) of SMA type 3 patients at baseline (Pre) and after five injections (Post) of Nusinersen (6‐month follow‐up) (n = 12); four patients had undetectable pNfH levels both at baseline and during follow‐up, while two patients displayed a similar baseline value of NfL (201.1 and 202.7 pg/mL) that became undetectable in both cases at the end of the follow‐up. Correlation between the change in pNfH (B) or NfL (D) values and variations in HFMSE scores over time (n = 12); two patients displayed identical differences in HFMSE scores (4 points) and differences in pNfH levels (0 ng/mL)

Interestingly, the comparison between the values of both neurofilament types before and after 6 months from treatment start showed a slight but significant decrease (pNfH: median of differences −0.0236 ng/mL, *P* = .016—NfL: mean of differences −70.0 ± 97.7 pg/mL, *P* = .031) (Figure 3A and 3). However, no significant correlation was observed between the pre‐post–difference in HFMSE scores and the change of either pNfH or NfL over time (Figure 3B and 3).

Baseline values of both pNfH and NfL in the patient with SMA type 1 were much higher than those observed in healthy controls (6.874 ng/mL and >10 000 pg/mL, respectively), in accordance with existing data from the literature.^7^, ^12^ Levels of both neurofilament types showed a progressive and rapid reduction, and at 6 months from treatment start they were comparable to those of healthy individuals (data not shown).

## 4 DISCUSSION

In this explorative study on a small cohort of SMA type 3 patients, we report our preliminary findings on the effects of Nusinersen administration on motor function over the short‐term and describe changes over time in the CSF concentration of both pNfH and NfL, which were assessed as potential biomarkers of treatment response.

At 6 months from treatment start, we observed a slight improvement in motor functions of treated patients, which, however, did not reach nominal significance compared to pre‐treatment values. A recent post hoc analysis of data from the CS2/CS12 study, which included a subset of ambulant patients with SMA type 2 and 3 treated with Nusinersen, showed an improvement in motor performance (measured by the 6MWT) at 35 months from treatment start.^13^ The apparent inconsistence with our results is probably due to the significantly different follow‐up extension, to baseline patient characteristics and disease duration. In our study, we selectively recruited SMA type 3 patients, who were older compared to the CS2/CS12 cohort (64% of patients included in the study by Montes et al were 11 years old or less). None of our patients was treated in the initial stages of the disease, and disease progression was slow for all of them. Milder phenotype and older age could explain the less striking effect of Nusinersen over the short follow‐up of our study.

Neurofilaments in the CSF of affected patients at baseline were comparable to age‐matched controls and were all consistently below the threshold values that usually define pathological samples.^14^ This finding possibly reflects the slow disease progression typical of SMA type 3, in which axonal degeneration is a smouldering process that is less likely to significantly affect neurofilament concentration. As previously described for healthy individuals,^15^ NfL concentration was positively correlated with age and duration of the disease in SMA type 3 patients, while pNfH levels were less influenced by patient age and disease duration.

Near‐normal baseline values notwithstanding, the CSF concentration of both pNfH and NfL was significantly reduced at 6 months compared with pre‐treatment values. This finding might suggest an early biochemical effect of treatment, possibly reflecting decreased axonal degeneration, which may be captured well before any change in motor performance becomes clinically evident.

Similarly to our study, Wurster and colleagues^16^ have elegantly described the changes of CSF parameters over time in a cohort of SMA type 2 and 3 patients treated with Nusinersen. In their report, however, no significant difference in neurofilament concentrations was found between pre‐ and post‐treatment samples. Our study design may have granted us more power to detect significant changes owing to its longer follow‐up (6 months compared with 2 months after treatment start) and to the cohort restricted to SMA type 3 patients. In addition, patients enrolled in our study had an overall shorter disease duration, which may have further affected results. Notably, some of the variability in results could be potentially related to difference in sensitivity among ELISA kits employed, especially for pNfH levels.

Despite these encouraging results, we did not observe any significant correlation between neurofilament concentration over time and the change in motor functions at six months as assessed by the HFMSE score. We speculate that the lack of a significant clinical effect during this relatively short follow‐up may in part explain this inconsistency.

Indeed, important limitations of our study include the small number of patients and the relatively short duration of follow‐up. In addition, analytical limitations need to be discussed: the baseline concentration of neurofilaments in our study was below the lower limit of quantification in a significant proportion of patients (4/12 and 2/12 for pNfH and NfL, respectively), thus hindering data interpretation. However, the remarkable consistency of measurable data and the significant differences observed despite such limitation, infers against a casual effect. These concerns may be addressed in future by the use of more sensitive methods designed to detect lower analyte concentrations, such as the single‐molecule array immunoassay that has been recently developed for NfL detection.^17^

Overall, our studies suggest that both pNfH and NfL are reduced by Nusinersen administration in a significant proportion of SMA type 3 patients as early as six months after the first treatment, and mandate further investigation on the accuracy of neurofilaments as markers of treatment response.

## CONFLICTS OF INTEREST

The authors confirm that there are no conflicts of interest.

## AUTHOR CONTRIBUTIONS

AG, SC, FM, IF, GR, MM, EA, DV, GC and EP visited the patients, performed CSF collection and drug dispensation. DS, DR, VP and LP performed laboratory assessment. IF and AG analysed the data and wrote the manuscript. All authors read and approved the final version of the manuscript.

## Data Availability

The data that support the findings of this study are available from the corresponding author upon reasonable request.
